# Intraprocedural Angiographic Lung Perfusion: A Novel Application for Real-Time Integration Into a Fluoroscopic Platform

**DOI:** 10.1016/j.jscai.2026.105617

**Published:** 2026-08-20

**Authors:** Arash Salavitabar, Daniel Gruenstein, Chance Alvarado, Andrew Kuhls-Gilcrist, Gurumurthy Hiremath

**Affiliations:** a The Heart Center, Nationwide Children’s Hospital, Columbus, Ohio; b Pediatric Cardiology, Northern Light-Eastern Maine Medical Center, Bangor, Maine; c Center for Biostatistics, The Ohio State University, Wexner Medical Center, Columbus, Ohio; d Biostatistics Resource, Abigail Wexner Research Institute, Nationwide Children’s Hospital, Columbus, Ohio; e Canon Medical Systems USA, Tustin, California; f Division of Pediatric Cardiology, Department of Pediatrics, University of Minnesota Masonic Children’s Hospital, Minneapolis, Minnesota

**Keywords:** angiography, branch pulmonary artery stenosis, congenital heart disease, lung perfusion

## Abstract

**Background:**

Assessment of differential lung perfusion is currently limited to periprocedural lung perfusion scans (LPS) or magnetic resonance imaging (MRI), which do not enable for real-time decision making. This study demonstrates the development and evaluation of a novel angiographic lung perfusion (ALP) technique in patients with congenital heart disease.

**Methods:**

The ALP technique was used to analyze differential branch pulmonary artery (PA) flow in retrospectively identified PA angiograms with corresponding LPS or MRI. Intraclass correlation coefficient and agreement were assessed. Two mathematical models were tested (area under curve and slope) at 2 time points (second peak and average).

**Results:**

Twenty-five angiograms were analyzed. The most common cardiac anatomy was tetralogy of Fallot, and most common indication for catheterization was unilateral branch PA stenosis. The slope-based methods demonstrated high intraclass correlation coefficients with LPS/MRI (ICC, 0.84-0.86), indicating good reliability (ranging moderate-to-excellent), whereas Bland-Altman analysis showed low overall bias.

**Conclusions:**

This ALP quantification application enables accurate lung perfusion assessment with potential for real-time integration into a fluoroscopic platform. This method provides a potential method for quantifying clinically meaningful intraprocedural information, particularly in assessing relative changes in differential perfusion before and after PA interventions. Further prospective studies are needed to confirm accuracy and clinical utility in broader populations.

## Introduction

Proximal branch pulmonary artery (BPA) stenosis is commonly observed in congenital heart disease (CHD).^1^^,^^2^ It may present as an isolated condition, as part of a broader pattern of right ventricular outflow tract obstruction, or, more frequently, in postoperative anatomy and in association with specific syndromes.^3^^,^^4^ A comprehensive evaluation of BPA stenosis severity and its hemodynamic implications involves assessing symptoms, performing detailed imaging for anatomical analysis, and using differential pulmonary blood flow (PBF) measurement along with cardiopulmonary exercise testing for functional assessment.^4^^,^^5^ Catheter-based interventions to relieve pulmonary artery (PA) obstruction are common in congenital and structural catheterization laboratories and use the degree of disturbance in normal PBF distribution as a key factor in determining not only whether there is an indication to perform an intervention to alleviate BPA obstruction but also procedural success.

Conventional assessments of differential PBF must be performed either before or after catheterization. Lung perfusion scans (LPS) use radionuclide and include additional radiation exposure.^6^^,^^7^ Increasingly, magnetic resonance imaging (MRI) and velocity mapping have been used to assess differential PBF and PA anatomy in CHD, thereby reducing the radiation burden and demonstrating accuracy comparable with LPS.^6^^,^^8^ There is no current commercially available mechanism for quantitatively assessing differential BPA flow during cardiac catheterizations. Patients must undergo preprocedural or postprocedural LPS or MRI for this purpose, which can involve additional radiation, sedation, and costs, while limiting the capacity for dynamic, intraprocedural decision making. Implementing intraprocedural lung perfusion quantification would enhance procedural decision making in complex congenital cardiac interventions. To address these challenges, we aimed to develop and evaluate a novel application for real-time angiographic lung perfusion (ALP) quantification (Central Illustration) that can be integrated into the Canon fluoroscopic platform (Canon Medical Systems USA).

**Central Illustration:**
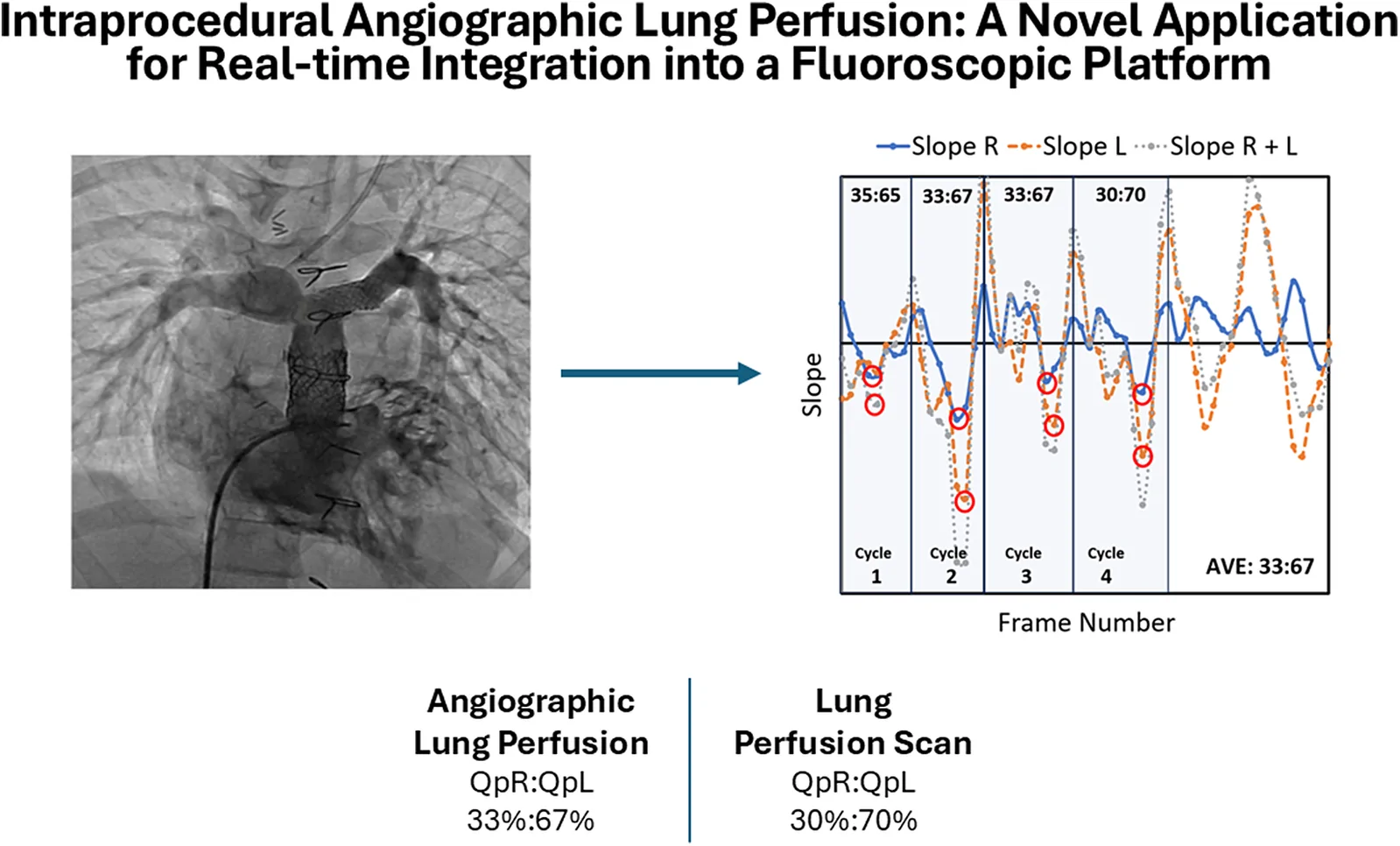
**Depiction of angiographic lung perfusion from angiogram acquisition to analysis to data, with an example of a comparative lung perfusion scan result for this patient.** QpL, left-sided pulmonary blood flow; QpR, right-sided pulmonary blood flow.

## Materials and methods

### Study design

This is a retrospective, 2-center study designed to investigate novel methods of quantifying differential BPA blood flow from transcatheter angiography. Institutional databases were reviewed to identify patients. The following inclusion criteria were used to identify angiograms performed using Canon (formerly Toshiba) interventional x-ray systems (platforms ranging from Infinix-i v4.2 software to Alphenix-i v9.0 software): (1) a cardiac catheterization that included power angiography within the main pulmonary artery or subpulmonary ventricle, (2) visualization of both lung fields and without significant overlap of BPA by non-PA vessels, and (3) differential PA perfusion quantification data from LPS or MRI performed within 6 months of pre-transcatheter pulmonary angiography or within 1 month post-transcatheter pulmonary angiography. Patients were excluded if a nonselective pulmonary angiogram was not performed, patients had sources of competitive PBF, patients had superior or inferior cavopulmonary circulations, or the consensus among the investigators was that the pulmonary angiography quality was not satisfactory (ie, lung fields were not adequately visualized within the frame or angulation of the image resulted in excessive overlap of lung fields). Baseline patient and procedural characteristics were collected.

### Ethical statement

This research was carried out in accordance with the appropriate ethical guidelines. This study was approved by each institutional review board and was exempt from the requirement of informed consent, with data protected to avoid ethical issues.

### ALP quantification methodology

The concept of a tracer-kinetic model is well described and uses an iodine contrast agent as a tracer rather than radioisotope particles as with LPS.^9^ Each image frame in an angiographic sequence corresponds to a time of *t* = frame number/frame rate, where frame rates were either 15 or 30 frames per second (fps). In this study, the ALP analysis was initiated with the identification of the frame in the angiographic sequence immediately prior to when the presence of contrast was first visible (*t* = *t*_1_).

The *t*_1_ image frame was subsequently used as a mask and log subtracted from all subsequent images, analogous to processes used for digital subtraction angiography, canceling signal inhomogeneities resulting from different underlying anatomical attenuation characteristics and patient motion, including respiratory motion. Following log subtraction, the subsequent pixel values represent the change in signal between each frame and the mask frame, and time-signal intensity curves (TIC) can be obtained on a frame-to-frame basis.

Regions of interest (ROI) were identified on the log-subtracted image sequence by a congenital interventional cardiologist using a polygon drawing tool over the right and left lung regions while excluding the heart shadow (Figure 1, top left). As the iodinated contrast is injected, enters the vasculature, and is observed in the imaging field of view, there is a decrease in signal resulting from increased attenuation of x-rays that is proportional to the volume of contrast media. The sum of the pixel values within the right (*S*_R_) and left (*S*_L_) ROI was calculated, corresponding to the total volume of iodinated contrast within the right and left lung at time *t*, for each image frame from the first presence of contrast (*t*_1_) to just prior to levophase (*t*_levo-1_). Values were recorded for 2 to 6 cardiac cycles between *t*_1_ and *t*_levo-1_, depending on variable heart rates and angiographic injection conditions, resulting in the TIC. Figure 1 (top right and bottom right and left) is an example of an angiogram with 5 cardiac cycles.

**Figure 1:**
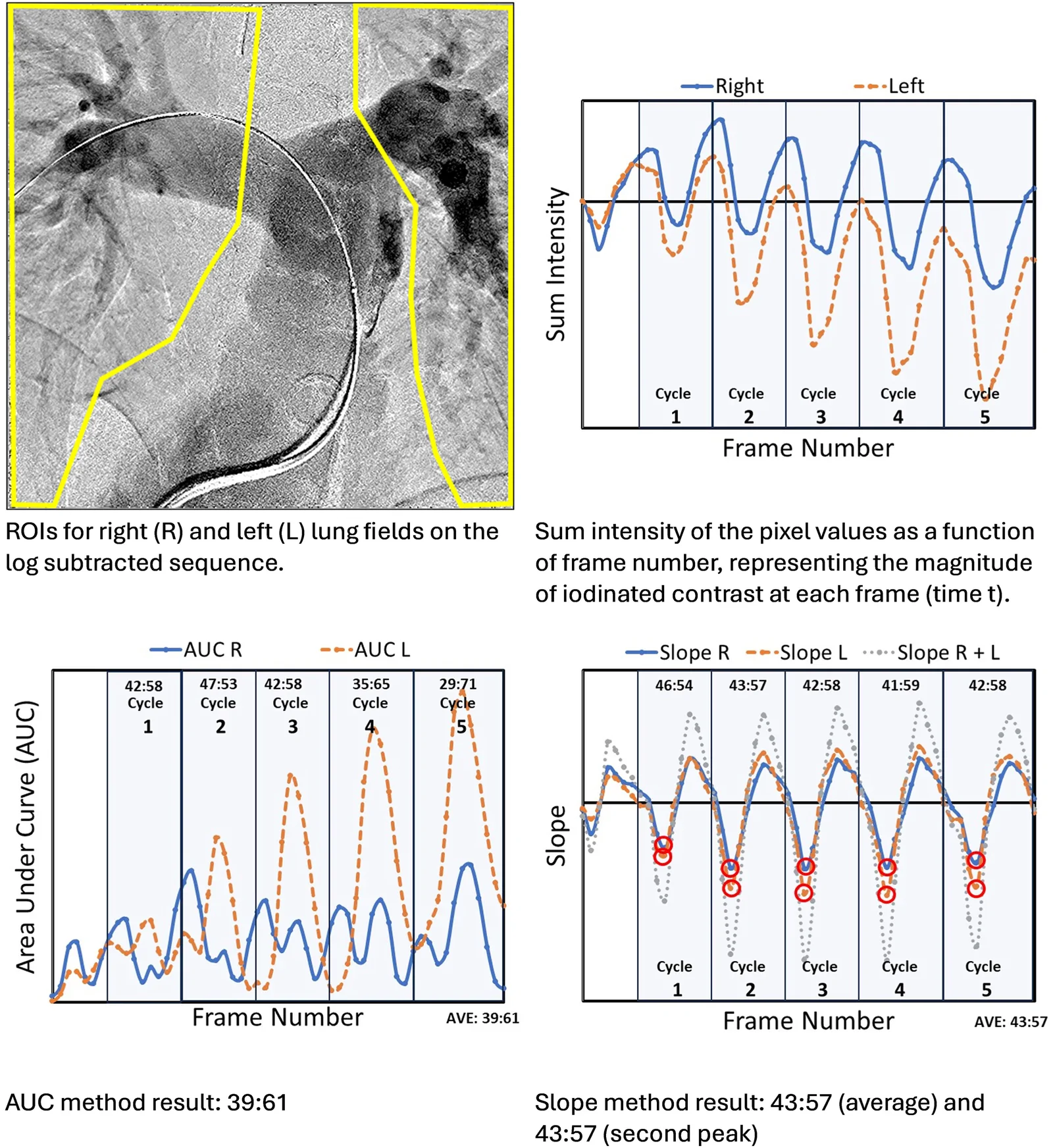
**Example of selection of ROIs on the log-subtracted angiographic image (top left), followed by the sum intensity of pixel values as a function of frame number over 5 cardiac cycles (top right), and the AUC(bottom left)and slope(bottom right)method analyses of ALP for a single patient.** ALP, angiographic lung perfusion; AUC, area under the curve; ROI, region of interest.

Two different methods were used for determination of the ALP as follows: (1) area under the curve (AUC) method and (2) slope method. For each method described, ALP calculations were performed at 2 time points: (1) the second cardiac cycle of the angiogram, in order to capture the time in which the greatest amount of contrast was likely to fill the cardiac structures without significant washout from noncontrast blood flow, and (2) the average over all cardiac cycles within the *t*_1_ to t_levo-1_ timeframe, in order to not only compensate for the variability in timing of contrast flow into a stenotic BPA but also account for washout from noncontrast blood flow.^9^

#### AUC method

The AUC was calculated over each cardiac cycle between *t*_1_ and *t*_levlo-1_ using the following formula:

$${\text{AUC}}_{R}=\sum_{f=1}^{n-1}\frac{S_{R}\left(f\right)+S_{R}\left(f+1\right)}{2}$$

$${\text{AUC}}_{L}=\sum_{f=1}^{n-1}\frac{S_{L}\left(f\right)+S_{L}\left(f+1\right)}{2}$$

where *S*_R_ and *S*_L_ are the sums of pixel values in the right and left ROI and *f* is the frame number with *f* = 1 and *f* = *n* representing the first and last frame for each cardiac cycle, respectively. The ratio of AUC_R_ to AUC_L_ for the second cycle and average of all cycles was then determined.

#### Slope method

The slope of a regression line of the TIC were calculated over a time window of 200 milliseconds, which is the approximate time when contrast agent first arrives at the first branch of the PA to the time when contrast agent fills the entire lung field during the torrent period while allowing for a sufficient minimum number of data points to be used in the calculation.^10^ This corresponds to 3 frames for 15 fps or 6 frames for 30 fps acquisitions. The formula for a 30-fps acquisition is as follows:

$${\text{Slope}}_{R}\left(f_{n}\right)=\frac{\sum_{f=n}^{n+5}\left[t\left(f\right)-\bar{t}\right]\left[S_{R}\left(f\right)-\bar{S_{R}}\right]}{\sum {\left(t\left(f\right)-\bar{t}\right)}^{2}}$$

$${\text{Slope}}_{L}\left(f_{n}\right)=\frac{\sum_{f=n}^{n+5}\left[t\left(f\right)-\bar{t}\right]\left[S_{L}\left(f\right)-\bar{S_{L}}\right]}{\sum {\left(t\left(f\right)-\bar{t}\right)}^{2}}$$

For a 15-fps acquisition, the slope of a regression line would span 3 frames (ie, *f* = *n* to *n* + 2). The maximum slope was identified in each cycle, and a ratio of the maximum Slope_R_ and Slope_L_ for the second cycle and average of all cycles was then determined.

### Statistical analysis

The intraclass correlation coefficient (ICC) was used to quantify the reliability of experimental modalities in reference to the gold standard perfusion/MRI modality. ICC was derived from a 2-way mixed-effects model assessing absolute agreement.^11^ ICC was presented alongside 95% CI and was interpreted as follows: <0.5, poor reliability; 0.5 to 0.75, moderate reliability; 0.75 to 0.9, good reliability; and >0.9, excellent reliability.^12^

Agreement was additionally assessed via Bland-Altman plots with differences among the y-axis and the mean of compared modalities along the x-axis, as recommended by Bland and Altman.^13^ For consistency, the right pulmonary artery values were used for all analyses. Overall bias was defined as the mean difference between modalities, and 95% limits of agreement were generated from the mean difference ± 1.96 times the SD of the difference. Univariable linear regression models were implemented to assess proportional bias (ie, differential levels of bias for varying magnitudes of measurement), with slope estimates presented alongside 95% CI and associated *P* values.^14^ All statistical analyses were performed using R version 4.2.2 (R Core Team).

## Results

### Patient characteristics

There were 25 angiograms that met inclusion criteria in a total of 22 patients. Patient and procedural characteristics are shown in Table 1. BPA stents were present in 32% (n = 8) angiograms. Cardiac anatomy varied, with tetralogy of Fallot being most common (36%). The most common indication for catheterization was BPA stenosis (92%).

**Table 1:** Patient and procedural characteristics

Characteristics	Value
No. of patients	22
No. of angiograms	25
Angiograms per center	
Center 1	6 (24)
Center 2	19 (76)
Absolute C-arm angulation, °	
LAO/RAO	9 (0-22)
CRA/CAU	30 (16-32)
Age, y	5.0 (1.0-11.0)
Weight, kg	20.8 (10.5-58.1)
BSA, m^2^	0.9 (0.5-1.7)
Cardiac anatomy (n = 25)	
TOF s/p repair	9 (36)
d-TGA s/p repair	6 (24)
TOF/APV	3 (12)
LPA stenosis s/p surgical PDA ligation	2 (8)
Isolated LPA s/p repair	2 (8)
Native BPA stenosis	1 (4)
Truncus arteriosus s/p repair	1 (4)
Congenital valvar pulmonary stenosis s/p BPV	1 (4)
Indications for catheterization	
Unilateral BPA stenosis	15 (60)
Bilateral BPA stenosis	8 (32)
Conduit stenosis	2 (8)
Transcatheter pulmonary valve implantation	2 (8)
BPA stent present	8 (32)

Values are n (%) or median (IQR).

APV, absent pulmonary valve; BSA, body surface area; BPA, branch pulmonary artery; CAU, caudal; CRA, cranial; d-TGA, d-transposition of the great arteries; LAO, left anterior oblique; LPA, left pulmonary artery; PDA, patent ductus arteriosus; s/p, status post; RAO, right anterior oblique; TOF, tetralogy of Fallot.

### Lung perfusion analysis

Scatter plots of the ALP vs LPS/MRI measurements for each angiogram are shown in Figure 2. Figure 3 demonstrates an example of a patient’s transcatheter angiography, LPS, and ALP analysis as a visual comparison. The overall agreement between LPS/MRI and the angiographic flow quantification modalities is shown in Table 2. The slope second peak showed the greatest ICC and lowest SD of the difference from LPS/MRI values, and the slope average modality showed the smallest average difference from LPS/MRI values. ICC was comparable for slope average and slope second peak modalities, both showing good agreement with the 95% CI ranging from moderate-to-excellent agreement. AUC-based methods showed moderate agreement, with the 95% CI ranging from poor-to-good agreement. On average, AUC and AUC second peak methods yielded lower RPA flow values than LPS/MRI, and the slope average and slope second peak methods resulted in higher RPA flow values than LPS/MRI. Variability in the differences between ALP measurements and LPS/MRI was less for slope methods, when compared with AUC-based methods.

**Figure 2:**
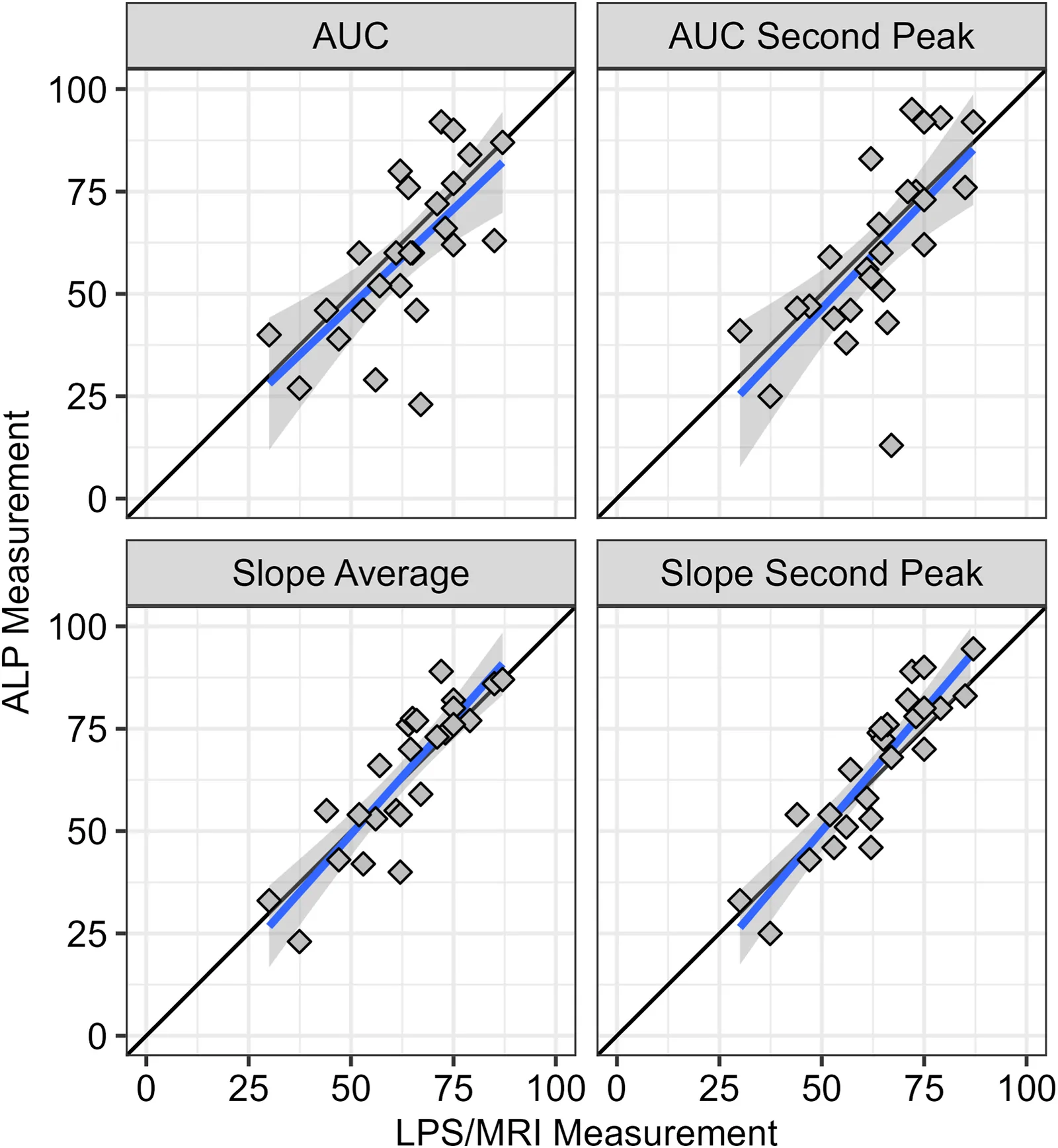
**Scatter plots for each ALP technique.** The blue line represents the linear trend of each dataset. The black line represents what would be a perfect correlation. ALP, angiographic lung perfusion; AUC, area under curve; LPS, lung perfusion scan; MRI, magnetic resonance imaging.

**Figure 3:**
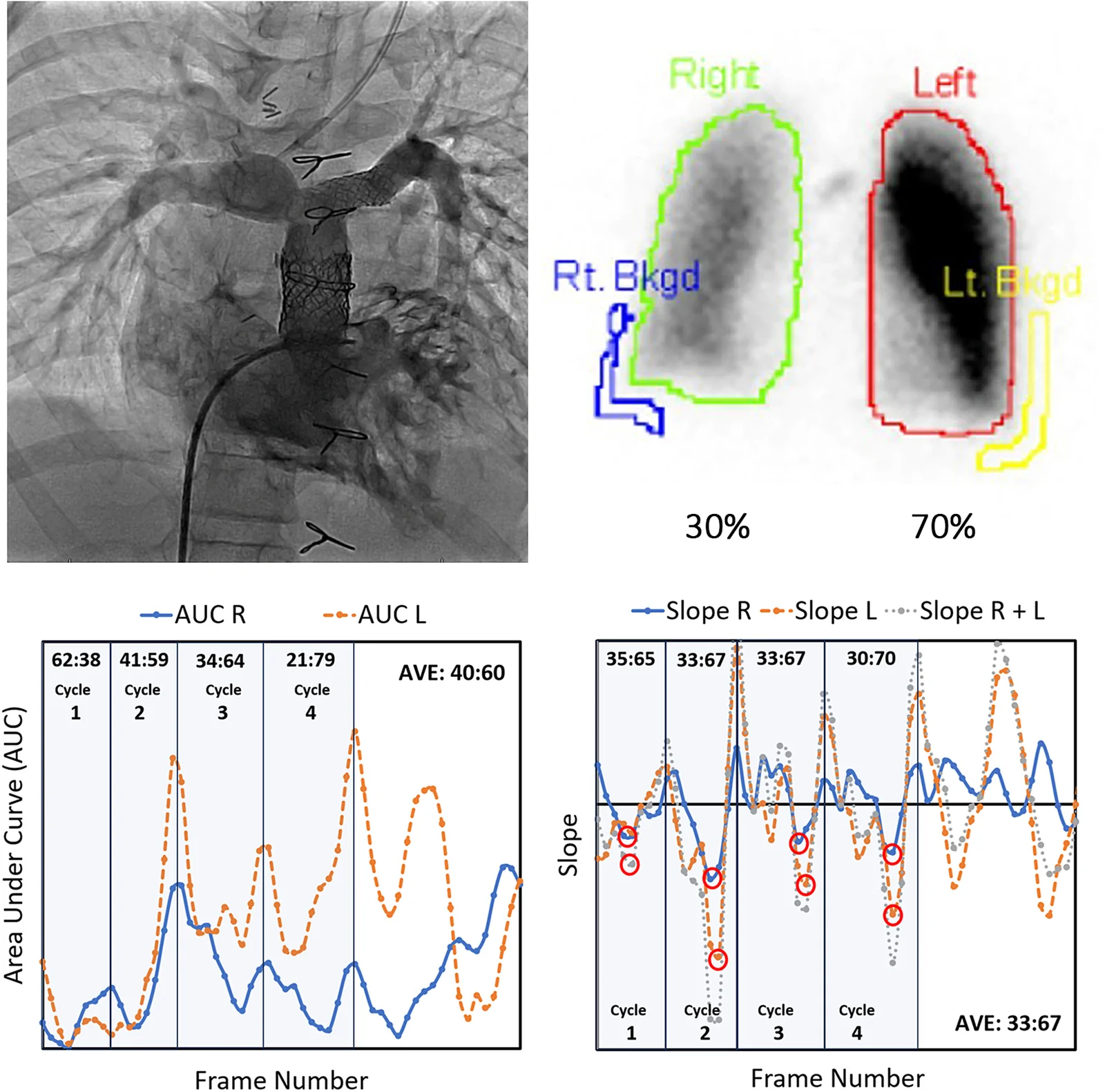
**Comparison of a patient’s transcatheter angiography (top left), lung perfusion scan (top right), and ALP analysis by the AUC (bottom left) and slope (bottom right) methods.** ALP, angiographic lung perfusion; AUC, area under the curve.

**Table 2:** Agreement between lung perfusion scan/MRI and modalities of quantifying angiographic lung perfusion

Modality	Measurement (right %)	Difference	ICC (95% CI)
LPS/MRI	63.2 ± 14.0	—	—
AUC	59.6 ± 19.7	3.6 ± 14.6	0.63 (0.33-0.82)
AUC second peak	60.3 ± 21.7	2.9 ± 16.0	0.62 (0.31-0.81)
Slope average	64.1 ± 18.1	−0.9 ± 9.2	0.84 (0.68-0.93)
Slope second peak	65.6 ± 18.4	−2.4 ± 8.6	0.86 (0.71-0.93)

Values are mean ± SD unless noted.

AUC, area under curve; ICC, intraclass correlation coefficient; LPS, lung perfusion scan; MRI, magnetic resonance imaging.

Figure 4 demonstrates the bias of the ALP quantification modalities alongside the upper and lower limits of agreement. The limits of agreement are the narrowest for slope second peak, consistent with its smallest SD (Table 2) and overall bias was least for the slope average. There was a proportional bias, in that for the higher average ALP measurements for the RPA, ALP gave greater values than LPS/MRI, while the opposite is true for lower average measurements. This proportional bias would create the opposite effect of proportional bias for the ALP to the LPA in this subject pool, in that ALP would provide lower values than LPS/MRI for the higher average ALP measurements. For the AUC and AUC second peak modalities, the slopes of the trend lines were estimated as −0.4 (95% CI, −0.77 to −0.04; *P* = .03) and −0.51 (95% CI, −0.87 to −0.15; *P* = .01), respectively. For slope average and slope second peak modalities, the slope estimates were −0.28 (95% CI, −0.5 to −0.05; *P* = .02) and −0.29 (95% CI, −0.49 to −0.09; *P* = .01), respectively.

**Figure 4:**
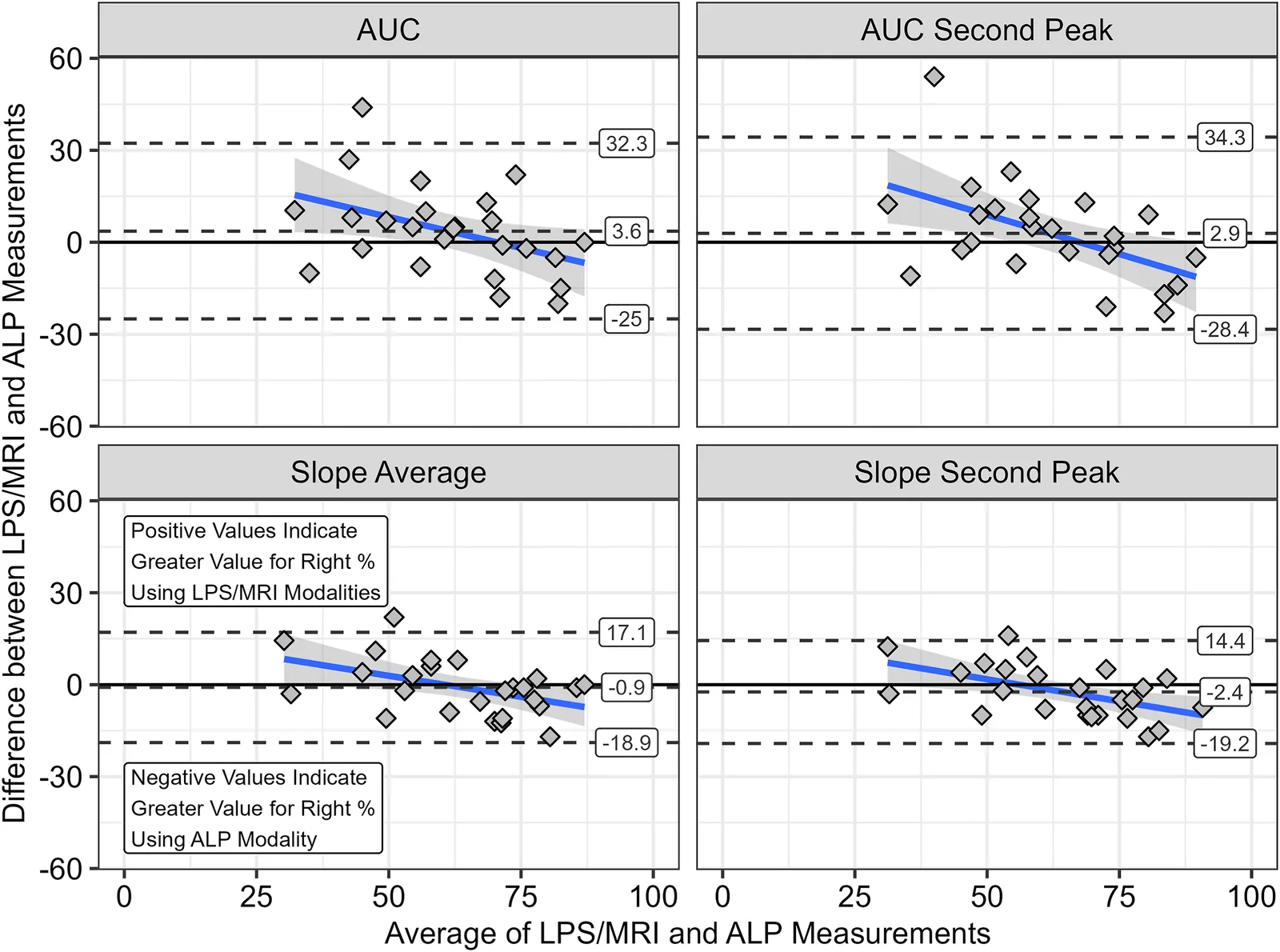
**Bland-Altman plots for each modality of quantifying ALP.** ALP, angiographic lung perfusion; AUC, area under curve; LPS, lung perfusion scan; MRI, magnetic resonance imaging.

## Discussion

The ALP application was designed to provide a real-time measurement of differential lung perfusion, which can be integrated into a fluoroscopic platform, with prior work demonstrating a processing time of <10 seconds.^10^ The intent of this study is to present an early-stage feasibility and validation analysis, demonstrating that ALP can provide quantitative estimates of differential BPA flow with meaningful agreement to established methods. Retrospective analysis of pulmonary angiography from 2 congenital cardiac catheterization laboratories showed good agreement with LPS/MRI measurements using slope-based ALP methods, with the 95% CI ranging from moderate-to-excellent agreement. These results are encouraging, despite the retrospective use of angiography, and are evidence that further validation studies are warranted. Moderate agreement was observed by AUC-based methods, with the 95% CI ranging from poor-to-good agreement.

Nuclear medicine perfusion imaging represents the current clinical reference standard for assessing differential lung perfusion due to its accessibility, rapid acquisition, and reproducibility. In this study, MRI-based measurements were included only when nuclear perfusion data were not available, reflecting the retrospective nature of the dataset. MRI-derived measurements represent PBF quantification rather than true perfusion imaging and are used as a secondary comparator. Phase-contrast MRI–derived PBF measurements have been previously shown to correlate well with radionuclide perfusion imaging in populations with CHD.^6^^,^^8^ Computed tomography–based perfusion techniques, including iodine mapping, are emerging approaches for assessing pulmonary perfusion.^15^ However, their clinical use in pediatric CHD remains limited due to radiation exposure and lack of standardized protocols.

The field of CHD has classically relied on LPS and MRI to quantify differential PBF to aid in the management of BPA lesions. The typical limitation of assessing and treating BPA stenosis in the cardiac catheterization laboratory is that real-time differential lung perfusion cannot be measured, either at baseline or directly following an intervention. This limitation results in the potential for delayed diagnosis, need for additional periprocedural studies, limited quantification of the degree of success of PA interventions prior to leaving the laboratory, and need for additional procedures. This burden accentuates the need for more advanced forms of intraprocedural assessment of lung perfusion.

Advanced forms of intraprocedural computational fluid dynamics have been demonstrated from transcatheter 3-dimensional (3D) rotational angiography of coarctation of the aorta^16^^,^^17^ but have not yet been demonstrated to measure differential flow in PA lesions. Attempts have been made to quantitatively assess PBF angiographically.^2^^,^^18^, ^19^, ^20^ Phillips SmartPerfusion software (Philips Healthcare) has been used to analyze flow changes, including time to peak, wash-in rate, washout rate, AUC, and mean transit time in adults with chronic thromboembolic PA stenosis undergoing balloon pulmonary angioplasty. This method does not offer differential blood flow measurement.^19^ Angiographic image processing to quantify differential flows in BPA and pulmonary vein stenoses associated with CHD has been explored using the fluoroscopic flow calculator, an open-source postprocessing tool developed in Python and integrated within Slicer Heart, a cardiac-focused extension of the 3D Slicer medical image computing platform.^2^^,^^20^ This approach used a calculation of the difference in pixel intensity from a contrast injection, when compared with baseline, and requires transferring the angiograms to a specific computing platform for analysis, a process that could be limited by processing time and ability to perform intraprocedural analysis.

The ALP application was designed to expand the rigor of calculating angiographic flow. ALP does not require selection of arterial input or venous output. ALP is based on analysis of TICs derived from contrast propagation within the lung fields following a single angiographic injection, consistent with previously described angiographic tracer-kinetic approaches.^9^^,^^10^ The relative distribution of contrast between right and left lung regions serves as a surrogate for differential PBF. Previous evaluation has demonstrated that the ratio of the signal intensity in the inflow phase of x-ray angiography within the lung fields (ie, ALP) is equivalent to the ratio of the signal intensity in lung scintigraphy in a small cohort.^9^ Although prior studies were performed under controlled gantry angles (eg, no right anterior oblique/left anterior oblique angulation), this study reflects, and is similarly limited by, real-world clinical angiography where patient positioning and gantry angulations must often be widely variable to demonstrate the desired anatomy. As a result, ROI were manually defined to ensure accurate representation of pulmonary vasculature, while excluding cardiac structures and overlapping anatomy. Despite being performed under nonstandardized conditions, there was good overall agreement with reference standards in our cohort. Future works will explore artificial intelligence (AI)–based approaches for automated vessel segmentation, ROI selection, and ROI standardization.

Log subtraction of the images was a crucial step in this study, as it would otherwise have introduced temporal variability in the underlying baseline pixel values, which would have differed across each of the ROI and potentially skewed the results significantly. The sum of pixel values within the ROI of each BPA was analyzed with the AUC and slope methods, which were further evaluated during the second cardiac cycle of the angiogram and during an average over all cardiac cycles. This multifaceted evaluation was conducted to identify the methodology with the greatest agreement with LPS/MRI, which identified the greatest agreement with the slope second peak and slope average methods. Additionally, the mean differences between the absolute values of LPS/MRI and these methods were clinically negligible in this small cohort. There was low overall bias, and importantly, there was proportional bias of angiographic flow measurements at more extreme LPS/MRI values, which will require further investigation in larger studies. The goal of ALP is not to replace LPS/MRI, but rather to provide intraprocedural, real-time estimation of differential perfusion, which may complement or reduce the need for additional postprocedural imaging or follow-up procedures in selected cases.

Angiography-based techniques offer the potential for higher spatial resolution perfusion assessment than nuclear medicine perfusion. In this initial feasibility study, we intentionally focused on right vs left lung segmentation as a clinically meaningful and relevant metric, particularly relevant to BPA interventions and consistent with established reference standards used in clinical decision making. A more granular (eg, lobar or segmental) analysis is an important future direction of investigation for feasibility, including the potential role of AI-based segmentation approaches.

### Limitations

The angiograms in this study were retrospectively performed; therefore, angiographic settings, injection locations, and gantry angles could not be standardized. Results remained encouraging despite this lack of standardization in operator practice. Furthermore, the retrospective design inherently limits control over physiologic and technical variables, including heart rate, injection timing, and frame rate. To account for these factors, multiple cardiac cycles were analyzed, both single-cycle and averaged measurements were evaluated, and 2 independent mathematical models were tested (AUC- and slope-based methods).

The angiograms were chosen based on retrospective determination of adequate visualization of lung fields and the absence of significant overlap of structures, as well as the presence of appropriate LPS or MRI perfusion data. Therefore, the generalizability of these ALP quantification methods may be limited. This pilot study consisted of a small cohort for the abovementioned reasons, and, therefore, we could not account for confounders, such as specific gantry angles, patient and vessel size, and presence of stents. These confounders will need to be further controlled in future prospective studies. These novel ALP quantification methods are not currently approved by the US Food and Drug Administration and should be further evaluated with larger, prospective studies with a standardized angiographic approach.

## Conclusions

This ALP quantification application enables accurate measurement of lung perfusion with the potential for real-time integration into a fluoroscopic system. This method has the potential to enhance procedural decision making and outcomes and to complement traditional assessments of periprocedural lung perfusion. Larger, prospective studies are needed to confirm its accuracy and clinical value across diverse populations. Future enhancements to this novel application might include automated PA ROI selection through AI, in place of manual methods.

## Peer review statement

Section Editor Gurumurthy Hiremath had no involvement in the peer review of this article and has no access to information regarding its peer review. Full responsibility for the editorial process for this article was delegated to Associate Editor Frank F. Ing.

## CRediT authorship contribution statement

**Arash Salavitabar:** Conceptualization, Data curation, Formal analysis, Investigation, Methodology, Validation, Visualization, Writing – original draft, Writing – review & editing. **Daniel Gruenstein:** Conceptualization, Data curation, Formal analysis, Investigation, Methodology, Supervision, Validation, Writing – original draft, Writing – review & editing. **Chance Alvarado:** Conceptualization, Formal analysis, Investigation, Methodology, Writing – original draft, Writing – review & editing. **Andrew Kuhls-Gilcrist:** Conceptualization, Data curation, Formal analysis, Investigation, Methodology, Resources, Software, Validation, Visualization, Writing – original draft, Writing – review & editing. **Gurumurthy Hiremath:** Conceptualization, Data curation, Formal analysis, Investigation, Methodology, Supervision, Validation, Writing – original draft, Writing – review & editing.

## Declaration of competing interest

Arash Salavitabar is a proctor for Renata Medical and reports research support from Renata Medical, Medtronic, Edwards Lifesciences, and Starlight. Daniel Gruenstein is a consultant for Canon Medical Systems. Chance Alvarado: none. Andrew Kuhls-Gilcrist is an employee of Canon Medical Systems USA, Inc. Gurumurthy Hiremath is a proctor and consultant for B. Braun and an ad hoc consultant for Abbott.
